# Abdominal testicular vessel distribution in human fetuses - basis for Fowler-Stephens surgery

**DOI:** 10.1590/S1677-5538.IBJU.2023.9909

**Published:** 2024-02-07

**Authors:** Tatiana S. C. G. Benzi, Carla M. Gallo, Anneliese Fortuna, Waldemar S. Costa, Francisco J.B. Sampaio, Luciano A. Favorito

**Affiliations:** 1 Universidade do Estado do Rio de Janeiro Unidade de Pesquisa Urogenital Rio de Janeiro RJ Brasil Unidade de Pesquisa Urogenital – Universidade do Estado do Rio de Janeiro – Uerj, Rio de Janeiro, RJ, Brasil

**Keywords:** Cryptorchidism, Gubernaculum, Fetus, Surgical Procedures, Operative

## Abstract

**Purpose:**

To analyze the histology and distribution of abdominal testicular vessels in human fetuses

**Patients and Methods:**

We studied 19 fetuses (34 testes) ranging in age from 12 to 19 weeks post-conception. The fetuses were evaluated regarding crown-rump length (CRL), total length (TL) and body weight immediately before dissection. Each testis was dissected and embedded in paraffin, from which 5 µm thick sections were obtained and stained with Masson's trichrome and Anti-CD31 antibody to quantify the vessels. The stereological analysis was carried out with the Image Pro and Image J programs, using a grid to determine volumetric densities (Vv). Means were statistically compared using the unpaired T-test (p<0.05).

**Results:**

The fetuses presented mean weight of 222.5g, mean CRL of 15.3 cm and mean TL of 23.2 cm. All testes were in the abdominal position. The mean percentage of vessels (Vv) in the upper portion of the testis was 7.6% (4.6 to 15%) and in the lower portion the mean was 5.11% (2.3 to 9.8%), with a significant difference (p=0.0001). In the analysis between the upper portion of the right and left testes (p=0.99) and in the analysis of the lower portion of the right and left testes (p=0.83), we did not observe significant differences.

**Conclusion:**

The upper portion of the abdominal testis in human fetuses had a higher concentration of vessels than the lower portion. These results suggest that manipulation of the lower end of the testis during Fowler-Stephens surgery should be avoided in order to preserve the collateral circulation.

## INTRODUCTION

The testes can be located in the abdomen, inguinal canal, or external inguinal ring (prepubic) in patients with cryptorchidism (1). The purpose of surgical treatment of undescended testes is to decrease the risk of testicular cancer or fertility issues and the placement of the testes in the scrotum without atrophy or recurrence. Surgical complications are more common in cases of testicular anomalies, such as small or short vessel testicles (2).

In cases where the testicles are not palpable, laparoscopic or open surgical exploration is necessary to identify the morphology and anatomical locations of the testes, vas deferens and testicular vessels and thus select the most appropriate surgical technique (3). Most orchidopexies can be performed through inguinal or even scrotal access. Laparoscopy is often used to diagnose non-palpable testes, by assessing testicular position if the testes are atrophic or absent (4, 5). Laparoscopy allows simultaneous performance of orchidopexy with high success rates (6, 7).

Two-stage Fowler-Stephens surgery is one of the surgical options to treat high abdominal undescended testis (8). The knowledge of testicular vascular anatomy is a key point in this procedure (9-11). We hypothesized that there are no differences in vessel distribution between the superior and inferior testicular portions. The objective of this study was to analyze the histology and distribution of testicular vessels during the human fetal period.

## MATERIAL AND METHODS

The study was approved according to the ethical standards of the hospital's institutional committee on experimentation with human beings (IRB number: 30706720.8.0000.5259).

We studied 34 testes obtained from 19 human male fetuses ranging in age from 12 to 19 weeks post-conception (WPC) during the period from January 2021 to October 2022. All the fetuses were obtained from the maternity service of our hospital with parental approval. The fetuses were macroscopically well preserved, without anomalies of the urinary and genital system, and fetuses with syndromes were excluded.

The gestational age of the fetuses was determined in WPC according to the foot-length criterion, which is currently considered the most acceptable parameter to calculate gestational age (12-15). The fetuses were also evaluated regarding crown-rump length (CRL) and body weight immediately before dissection. The same observer performed the measurements.

**Figure 1:**
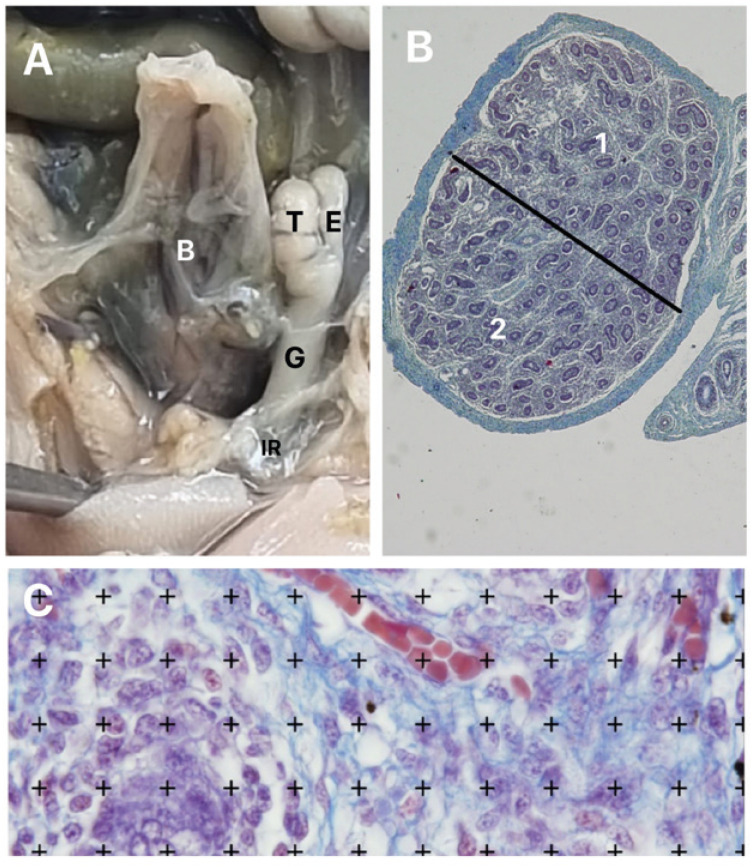
A) The figure shows a fetus with 17 weeks post conception (WPC) with the testis in abdominal position. After the dissection, the bladder (B), the testis (T), the gubernaculum (g), epididymis (e) and the internal inguinal ring (IR) can be observed; B) The photomicrograph shows the testis of a fetus with 16 WPC. A line was drawn, approximately in the middle part of the cut, dividing the testicle into upper (1) and lower half (1). Masson's trichrome X40; and C) morphometric analysis of the fetal testis. The photomicrograph shows the quantification of the vessels in a fetus with 16 WPC using the Image J Test grid software (Masson's trichrome X1000).

After the measurements, the fetuses were carefully dissected with the aid of a stereoscopic lens with 16/25X magnification. The abdomen and pelvis were opened to expose and identify the urogenital organs. Each testis and this respective genital mesentery were separated from the other structures and was fixed in 10% buffered formalin, and routinely processed for paraffin embedding, after which 5 µm thick sections were obtained at 15 µm intervals (Figure-1A).

The testicular vessels were studied by histochemical and immunohistochemical methods. The sections were stained with hematoxylin-eosin to assess the integrity of the tissue. We also performed the following staining: Masson's trichrome and CD-31 antibody (ABCAM- rabbit polyclonal anti- human and mouse CD31 with 1:50 dilution) to quantify and observe the testicular vessels (16).

Five sections were stained, and five fields of each section were selected. All selected fields were photographed with a digital camera (Olympus DP70, Tokyo, Japan) under the same conditions at a resolution of 2040 × 1536 pixels, directly coupled to the microscope (Olympus BX51, Tokyo, Japan). and stored in a TIFF file. A line was drawn approximately in the middle part of the cut, dividing the testicle into upper and lower half. The area density of blood vessels was calculated in each of the upper and lower parts (Figure-1B).

We used the Image J software, version 1.46r, loaded with its own plug-in to determine the vessels' volumetric density (Vv). Results for each field were obtained through the quantification assessment method, by superimposing a 100-point test grid (multipurpose test system) on the video monitor screen (Figure-1C). The arithmetic mean of the quantification in 5 fields of each section was determined (17, 18). Afterwards, we obtained the mean quantification value for the 5 sections studied from each testis (total of 25 test areas).

## Statistical Analysis

All parameters were statistically processed and graphically described. The Student t-test was used for comparison of quantitative data between upper and lower testicular portions The results obtained were compared using analysis of variance (ANOVA) with Tukey's multiple comparisons post-test (p<0.05). Simple linear correlations were calculated for all variables (where r^2^ values less than 0.4 reflect very weak correlation, r^2^ between 0.4 and 0.7 reflect moderate correlation and r^2^ greater than 0.7 indicate strong correlation): fetal weight, fetal age in post-conception weeks and percentage of vessel density in the upper and lower portions of the right and left testicles. The statistical analysis was performed with GraphPad Prism.

## RESULTS

The fetuses ranged in age from 12 to 19 WPC, weighed between 58 g and 551 g (mean=222.5 g), had CRL between 8 cm and 25 cm (mean=15.3cm) and total length between 11 cm and 56.5 cm (mean= 23.2cm). In 19 fetuses, all the 38 testes were in abdominal position. Table-1 reports all biometric data of the 19 fetuses in our sample.

**Table 1:** The tahle shows the biometric data of the fetus studied and the analysis of the vascular desinty in the 19 fetuses of our sample.

Fetus	WPC	RT UP Density (%)	RT LP Density (%)	LT UP Density (%)	LT LP Density (%)	Weight(g)	CRL (cm)	TL (cm)
1	12.9	7.3	3.6	6.0	4.0	58	8.0	11.0
2	13.1	6.6	4.4	5.7	4.7	66	15.0	21.5
3	13.1	9.8	5.0	10.0	6.3	70	11.0	14.0
4	13.8	x	x	6.4	3.8	80	14.0	17.0
5	13.9	8.6	6.8	15.0	9.8	107	17.5	21.0
6	14.7	7.8	4.8	7.0	4.5	170	15.0	18.5
7	16.0	5.5	3.8	x	x	130	15.5	22.0
8	16.0	7.2	5.4	5.3	2.3	246	18.9	24.7
9	16.0	8.0	4.3	5.5	3.5	286	18.2	24.2
10	16.1	5.6	7.4	7.0	4.3	192	8.5	18.0
11	16.3	5.0	4.8	7.5	5.5	247	13.5	18.5
12	16.3	10.0	5.0	5.8	3.3	250	17.0	23.0
13	16.5	7.0	5.4	6.3	3.3	230	17.5	25.0
14	17.0	5.8	3.3	8.2	7.6	348	14.0	56.5
15	17.4	x	X	7.6	8.6	252	18.0	24.5
16	17.6	6.5	4.0	9.0	6.0	250	16.5	24.0
17	18.1	9.2	5.0	4.6	4.4	214	11.5	20.0
18	19.1	4.8	3.3	12.0	6.2	481	17.0	25.0
19	19.4	14.2	9.8	x	x	551	25.0	32.5
**Mean**	**15.9**	**7.58**	**5.06**	**7.58**	**5.18**	**222.53**	**15.35**	**23.21**

WPC = weeks post conception; RT = Right testis; LT = Left testis; UP = Upper extremity of the testis; LP = Lower Extremity of the testis; CRL = crown rump length; TL = Total Length

**Figure 2:**
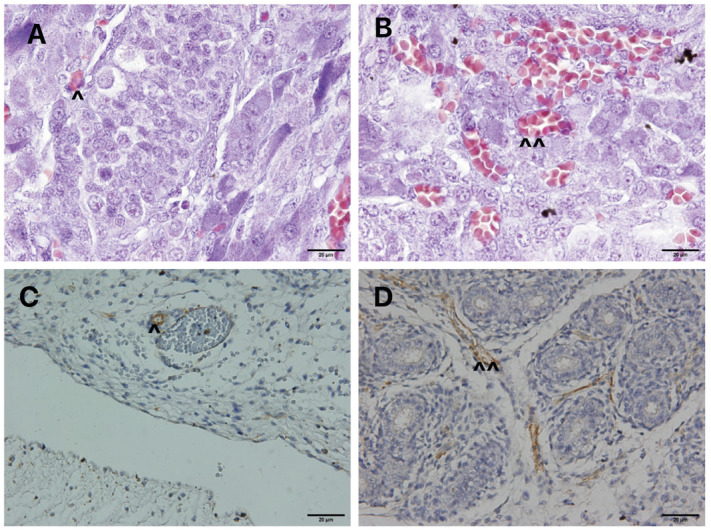
Histologic analysis of the testicular vessels.

During the histologic preparation we lost 4 testes, so we only analyzed 34 of the 38 testes. The statistical analysis of vessel volumetric density in the upper and lower portion of the testes studied is also reported in Table-1. In turn, Figure-2 shows the histological aspects of the vessel distribution in the upper and lower portions of the testes. The histological analysis showed that the upper pole had more vessels during the gestational period studied.

The quantitative analysis indicated no significant differences comparing the upper portion of the right and left testes (p=0.99) and in the analysis of the lower portion of the right and left testes (p=0.83), but the mean vessel volumetric density in the upper portion of the testes was 7.6% (4.6 to 15%, SD=0.42) and in the lower portion the average was 5.11% (2.3 to 9.8%, SD=0.31), with a significant difference (p=0.0001).

**Figure 3:**
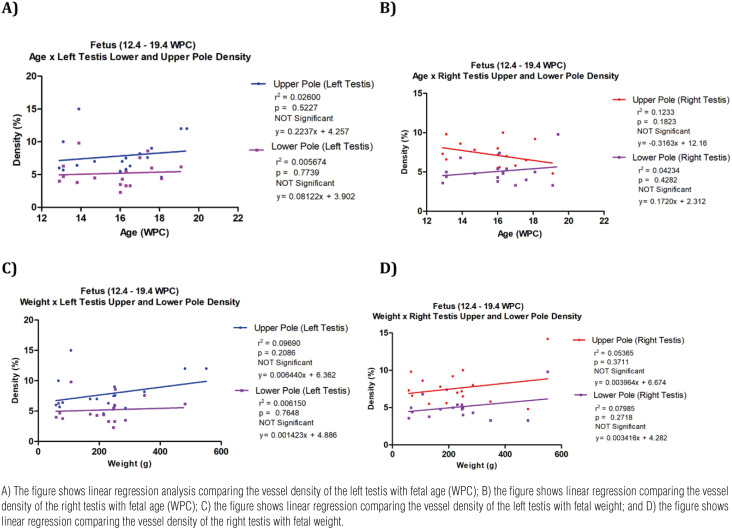
Linear regression analysis. The points plotted represent the mean values obtained for each week studied.

Linear regression analysis showed in the left testis that gestational age does not correlate with vessel density in the upper portion (y=0.2237x + 4.257; r^2^=0.02600; p=0.5227, not significant) and nor in the lower portion (y= 0.08122x + 3.902; r^2^=0.005674; p= 0.7739, not significant). In the right testicles, gestational age also did not correlate with vessel density in the upper portion (y= -0.3163x + 12.16; r^2^=0.1233; p= 0.1823, not significant) and nor in the lower portion (y=0.1720x + 2.312; r^2^= 0.04234; p = 0.4282, not significant) (Figure-3).

Linear regression analysis, in the left testis, showed that fetal weight did not significantly correlate with vessel density in the upper portion (y=0.006440x + 6.362; r^2^=0.09690; p=0.2086, not significant), nor in the lower portion (y=0.001423x + 4.886; r^2^=0.006150; p=0.7648, not significant). In the right testis, fetal weight also did not significantly correlate with vessel density in the upper portion (y= 0.003964x + 6.674; r^2^=0.05365; p=0.3711, not significant) nor in with vessel density in the lower portion. (y =0.003416x + 4.282; r^2^=0.07985; p=0.2718, not significant) (Figure-3).

## DISCUSSION

High abdominal undescended testis is a challenging situation in pediatric urology. Irrespective of the surgical technique, preservation of an adequate arterial supply for the testes is crucial for successful orchidopexy to ensure normal testicular size and texture (19). The anatomy of testicular arteries is very important to the success of the surgical procedures (11, 20). Each testis is irrigated by three arteries: the testicular artery (internal spermatic artery), a branch of the aorta; deferential artery (vasal artery), a branch of the inferior vesicle artery that originates from the anterior trunk of internal iliac artery and cremasteric artery (external spermatic artery), a branch of the inferior epigastric artery. These three arteries penetrate the organ in the mediastinal region, where they provide ample communication (11, 21).

The majority of vessels ending to the testis, regardless of their origin, enter through the testis hilum and hence the upper testis (11, 21). The anastomoses between the testicular, deferential and cremasteric arteries occur outside the testis (21, 22). The cremasteric anastomoses possibly occur via the epidiymal branch of the testicular artery and gubernaculum vessels what makes gubernaculum preservation important to maximise collateral flow to the testis during Fowler Stephens orchidopexy (11).

When orchidopexy is performed in patients with intra-abdominal testicles, one of four surgical techniques is applied: conventional orchidopexy, laparoscopic orchidopexy, Fowler-Stephens orchidopexy in one stage, and Fowler-Stephens orchidopexy in two stages (23). In the four types, surgery consists of locating each testis, dissecting it near the spermatic cord, acquiring free tension, and then repositioning it near the scrotum. When performed open, a medial inguinal incision is made from the anterior superior iliac spine to the external oblique fascia to allow exploration of the peritoneal cavity (24). Advantages of performing these techniques by laparoscopy include improved visualization, more extensive vascular dissection ability to vessel origin, lower morbidity, and ability to create a medial inner ring to the lower epigastric vessels and perform a straight course to the scrotum (25).

Conventional laparoscopic Fowler-Stephens orchidopexy (CLO), both in one and two stages, is adopted when the testes are located high, and the testicular vessels are too short to be fixed in the scrotum. The vessels are ligated and the blood supply to the testes is preserved via collateral circulation between testicular and deferential arteries, followed by repositioning and fixation of the testes (8, 26). In this surgery, the gubernaculum is divided, and each testis is brought down medially to the inferior epigastric vessels and a straight course to the scrotum is performed (4, 5).

CLO is an accepted technique when the spermatic vessels' length is short and to perform tension-free testicular mobilization at the scrotum. CLO atrophy rates can be as high as 33% (25). In this technique, previous testicular surgery and vas abnormalities are factors associated with testicular atrophy (26-28).

Recently, an alternative of CLO with gubernaculum preservation and anatomical delivery of the testis through the internal inguinal ring was described, called gubernaculum-sparing laparoscopic orchidopexy (GLSO) (29). It improves the collateral blood supply to the testes by preserving the gubernaculum and cremasteric vessels, with less atrophy than in CLO (29).

A previous anatomical study of cadavers with undescended testes demonstrated that all testicles studied, including the undescended ones, had testicular, deferential and cremasteric arteries and good communication among the three arteries, with visible anastomotic channels between the testicular and deferential arteries (30). An interesting study in human fetuses showed that the incidence of extra-numerical arteries in fetuses with testicles located in the abdomen is rare, and in cases of surgery by the Fowler-Stephens technique, they would be maintained by the deferential and cremasteric arteries (30).

This article is the first to evaluate the histology of testicular vessels in human fetuses with the testes positioned in the abdomen and demonstrates a significant difference between the vascular density in the upper and lower ends of the testicle, which reinforces the importance of preserving the vessels that will give rise to testicular collateral circulation in the lower extremity. Our study demonstrates that vascular density was positively correlated with the age and weight of the studied fetuses. Our results also show that the lower region of the testes has a significantly lower vascular concentration and corroborates the importance of preserving the vascularization that reaches the testes through the region of the testicular gubernaculum and the cremasteric vessels. As described for GSLO, this can be of great benefit and explain the lower atrophy rates observed with this technique (30).

Some limitations of our study should be mentioned: (a) the sample size was small (however, fetuses are rare, so observations of small samples are still relevant); and (b) the ideal sample for the present study would be children aged less than 1 year with cryptorchidism, but due to the rarity of this ideal sample, the study with human fetuses can provide important information for the treatment of cryptorchidism.

We concluded that the upper portion of the abdominal testis in human fetuses had a higher concentration of vessels than the lower portion. These results suggest that manipulation of the lower end of the testis during Fowler-Stephens surgery should be avoided in order to preserve the collateral circulation.
